# Gene Flow and Genetic Structure Reveal Reduced Diversity between Generations of a Tropical Tree, *Manilkara multifida* Penn., in Atlantic Forest Fragments

**DOI:** 10.3390/genes12122025

**Published:** 2021-12-20

**Authors:** Zubaria Waqar, Ramiris César Souza Moraes, Maíra Benchimol, José Carlos Morante-Filho, Eduardo Mariano-Neto, Fernanda Amato Gaiotto

**Affiliations:** 1 Applied Ecology Conservation Lab, Department of Biological Sciences State, University of Santa Cruz, Ilhéus 45662-900, Brazil; zubariawaqar@yahoo.com (Z.W.); mbsouza@uesc.br (M.B.); jcmfilho@uesc.br (J.C.M.-F.); 2 Genetics and Biotechnology Center, Department of Biological Sciences State, University of Santa Cruz, Ilhéus 45662-900, Brazil; ramiris.moraes@gmail.com; 3 Biology Institute, Federal University of Bahia (UFBA), Salvador 40170-115, Brazil; marianon@gmail.com

**Keywords:** biodiversity, molecular ecology, conservation, fragmentation, microsatellites

## Abstract

The Atlantic Forest remnants in southern Bahia, Brazil, contain large tree species that have suffered disturbances in recent decades. Anthropogenic activities have led to a decrease in the population of many tree species and a loss of alleles that can maintain the evolutionary fitness of their populations. This study assessed patterns of genetic diversity, spatial genetic structure, and genetic structure among *Manilkara multifida* Penn. populations, comparing the genetic parameters of adult and juvenile trees. In particular, we collected leaves from adults and juveniles of *M. multifida* in two protected areas, the Veracel Station (EVC) and the Una Biological Reserve (UBR), located in threatened Atlantic Forest fragments. We observed a substantial decay in genetic variability between generations in both areas i.e., adults’ H_O_ values were higher (EVC = 0.720, UBR = 0.736) than juveniles’ (EVC = 0.463 and UBR = 0.560). Both juveniles and adults showed genetic structure between the two areas (θ = 0.017 for adults and θ = 0.109 for juveniles). Additionally, forest fragments indicated an unexpectedly short gene flow. Our results, therefore, highlight the pervasive effects of historical deforestation and other human disturbances on the genetic diversity of *M. multifida* populations within a key conservation region of the Atlantic Forest biodiversity hotspot.

## 1. Introduction

Tropical forests retain the greatest biodiversity on Earth, yet they have been drastically deforested and degraded due to anthropogenic activities [1,2]. Currently, one-half of tropical and sub-tropical forests have been altered [3], and about 10% of forest area in all continents consists of fragments smaller than 10,000 ha [4]. The increased demand for timber, energy, food, and other agricultural products for human consumption is leading to intense deforestation, inducing global warming, and consequently contributing to biodiversity loss [3]. The remaining biota stranded in forest patches, especially those strictly associated with high-quality forest environments, is forecast to become locally extinct or suffer drastic population decline, with subsequent loss of diversity [5].

Understanding the effects of anthropogenic disturbances on ecological and evolutionary processes is vital to perceive the long-term viability of current populations, especially threatened and endemic ones. From this perspective, the importance is evident of integrating knowledge of the influence of habitat loss in a genetic approach to broaden the understanding of ecological and evolutionary processes. The population genetics approach allows inferences about connectivity between forest fragments by analyzing genetically distinct populations [6]. Estimations of genetic differences between populations can help to understand the evolutionary history and adaptive potential of species faced with forest loss and fragmentation [7]. Hence, a direct relationship exists between population dynamics and genetic diversity [8,9].

The decrease in species dispersion rate can affect the mating system and spatial genetic structure of trees. A decrease in species dispersion rate occurs because disconnection and increasing distance between populations increase the probability of mating between closely related individuals, leading to a reduction in genetic diversity due to inbreeding [10]. Additionally, a spatial genetic structure consisting of a nonrandom distribution of genotypes of certain species in a given space can be affected by natural selection, demographic history, and gene flow of species [11]. 

Since gene flow indicates the movement of genetic material between populations, its interruption increases the genetic divergence between populations, likely leading to their isolated among habitats [9]. Therefore, conservation strategies might be more effective by understanding the connectivity between forest fragments, including investigation of gene flow. Habitat loss can disrupt this gene flow, affecting breeding and dispersal success in plant assemblages in human-modified landscapes [12]. The negative impact on habitat connectivity due to anthropogenic activities can cause isolation of populations, with consequences on intercrossing between individuals and effective gene flow [13]. According to genetic variation dynamics, over space and time [14] a few genotypes of a population can become dominant over others due to natural selection, genetic drift, and inbreeding.

Additionally, inbreeding can generate drastic consequences for populations, such as increased homozygosity and decreased allele frequency, leading to allele fixation and inbreeding depression [15]. Genetic drift is one of the strongest evolutionary factors, since populations are composed of a finite number of individuals, and random allele frequency fluctuations are inevitable [16]. Small population size also increases the likelihood of allelic fixation due to inbreeding. As a result, it can reduce long-term viability.

It is challenging to understand the effect of the environment and human activities on species’ past and current genetic diversity [10,17]. Species’ evolutionary history is important to understand their capacity to resist stresses. In particular, the ability of species to cope with environmental disturbances is directly linked to their genetic diversity. Species with low genetic diversity likely have less chance to adapt to severely disturbed environments [10,18,19]. To ascertain the fitness of any individual genotype of a population and its evolutionary processes [20], it is necessary to quantify its survival and reproduction parameters. If changes in allele frequency are detected in a certain population due to anthropogenic activities, it is fundamental to understand whether they are caused by mechanisms such as migration, natural selection, and genetic drift [21]. The last two mechanisms can be related to human activities, such as hunting and fragmentation caused by logging or clearance for crops. In this scenario, estimation of genetic diversity parameters is a way to understand the human impact. As such, it is one of the maim challenges, particularly in small populations in tropical forests. To overcome this challenge, some authors [22] have published frameworks to detect genetic change between generations of a population by calculating allele frequency, to obtain detailed knowledge of gene flow within and between populations [23]. As mentioned before, analyses of genotypic frequencies also provide important information to understand the diversity and evolutionary processes between generations of certain species.

Molecular studies are widely used to provide insights on genetic structure, diversity, and relationships among species by investigating species at the population level. The Atlantic Forest in southern Bahia, Brazil, is a hotspot biome, including endemic and endangered species [24]. Due to anthropogenic disturbance, the original portion of the forest has been reduced to less than 10% [25,26]. Despite forming crucial areas of endemism, the Atlantic Forest fragments of southern Bahia have suffered long periods of anthropogenic disturbances of the biota. For example, [27] discussed the impact of human activities on species diversity of *Dalbergia nigra* (Vell.) Allemao ex Benth. Popularly known as Brazilian rosewood, it is an endemic Atlantic Forest species. Since 1998, it has been listed as “Vulnerable” due to overexploitation of trees for its high-quality timber. Some species affected by anthropogenic disturbances are likely to go extinct if they cannot adapt [5]. Despite forming an important area of endemism, the biota of Atlantic Forest remnants in southern Bahia has suffered the incidence of long periods of anthropogenic disturbances, mainly related to deforestation. Consequently, the populations of tree species such as brauna (*Melanoxylon brauna*), Bahia rosewood (*D. nigra*), and brazilwood (*Paubrasilia echinata*) have a declined [11,27,28].

According to previous studies [24,26], botanical surveys suggest that this region has the highest species richness per unit area and highly endemic plants. Endemic plants are generally adapted to a specific geographic area [29,30], which makes them prone to inbreeding due to isolation, so they generally have low genetic diversity [29,31]. Endemic plants have characteristics that make them vulnerable to anthropogenic disturbances, such as limited geographic distribution and small population size, ultimately leading to their local extinction [32,33].

Therefore, these endemic plant species have high global priority for preservation [30,32,33]. To achieve this, it is important to know whether the species has reduced genetic diversity or restricted gene flow due to small and isolated population [34,35]. Protected areas play a vital role in effective species conservation strategies [36], especially in the regions where endemic plant species are present, requiring extra biodiversity protection [37]. In this study, we assessed the genetic diversity of an endemic tropical tree, *M. multifida*, in two protected Atlantic Forest fragments in southern Bahia. Our focal tree species belongs to the genus Manilkara (Sapotaceae family), which is exploited for its hard and heavy wood, edible fruits, and latex. The fruits are consumed by a wide range of forest vertebrates, providing key food resources, especially for primates [38]. *M. multifida* is a large tree that can reach approximately 30 m in height, and only occurs in Bahia within remaining Atlantic Forest areas, classified as one of the most threatened biomes in the world. Due to the severe forest loss and fragmentation, the species is currently classified as “Endangered” by the International Union for Conservation of Nature (IUCN) [39], with remaining populations stranded in fragmented forest landscapes.

In this study, we (i) compared the genetic patterns among juvenile and adult trees within each protected area; (ii) estimated the genetic structure among *M. multifida* populations in the two protected areas; (iii) estimated the gene flow of this tree species within and between protected areas, to evaluate the dispersal distance. In particular, we predicted (i) lower genetic diversity of juveniles than adults, given they represent the generation under the most intense impact of forest loss and anthropogenic disturbances; (ii) a high level of genetic structure between populations due to the intense history of human disturbances in the region combined with the long distance between the two sampled protected areas, causing us to believe that both populations are derived from expansion from an original gene pool with consecutive migration events; (iii) short distance of gene flow of the populations due to isolation of the protected areas. Based on our findings, we finally discuss the effects of human disturbances on the loss of genetic diversity and genetic structure of *M. multifida* populations, comparing genetic parameters of adult and juvenile trees, to account for the period before and after forest loss.

## 2. Materials and Methods

### 2.1. Study Areas and Tree Sampling

**Figure 1:**
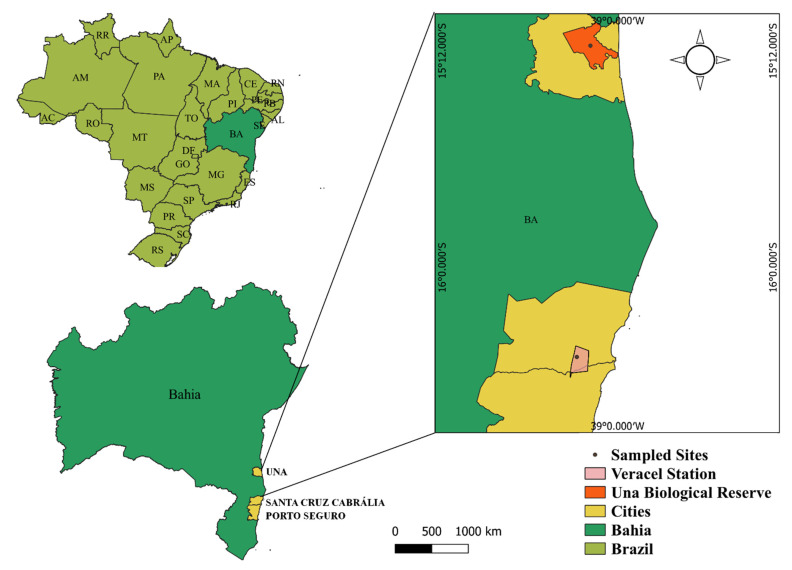
Study area, highlighting the Veracel Station (EVC) and Una Biological Reserve (UBR). Adapted from SOS Atlantic Forest Foundation/INPE. Geographic coordinate system Datum: WGS:84 Data Source: IBGE (2020) and Veracel Celulose (2021).

The Atlantic Forest is the second-largest tropical forest in South America. Its original area was approximately 1,500,000 km^2^, ranging along the Brazilian Atlantic coast, with additional patches in Argentina and Paraguay [40]. Currently, only 11.4% to 16% of its original area remains, distributed in fragmented landscapes [40]. We conducted the samplings in two protected areas located in the southern region of Bahia, Brazil—the Veracel Station (EVC) and the Una Biological Reserve (UBR), 145 km apart. The EVC was created in 1998 and retains a total area of 6069 ha, and UBR was established in 1980, encompassing 11,400 ha (Figure 1). Both protected areas are evergreen tropical forests and present similar floristic and vegetation structures, composed mainly of large and medium-sized trees, with a great abundance of lianas and epiphytes [41]. EVC has forest mostly of advanced age, considered a well-preserved Tabuleiro forest, with a uniform canopy typical of tropical lowland rainforests. The average temperature is 22.6 °C and mean annual rainfall is 919 mm [42]. UBR is embedded in human-modified landscapes, consisting of dense Ombrophylous forest. The region’s average annual temperature is 24 °C, and its mean yearly rainfall is 1600 mm [43]. 

We performed an active search for juveniles and adults of *M. multifida* in each protected area, keeping a minimum distance of 10 m from any sampled tree, since the genus is considered to be dispersed over short distances [44]. We considered trees to be adults with circumference at breast height (CBH) ≥ 70 cm, and juveniles with CBH ≤ 10 cm. We collected healthy young leaves from each individual and obtained the geographic coordinates using a GPS device (Garmin^®^, Schaffhausen, Switzerland). All told, we sampled leaves of 50 adults and 50 juveniles in EVC, and 45 adults and 34 juveniles in UBR. Our sampling corresponded to approximately 1/3 of the total area of EVC and 1/8 of the UBR area. The ratio between adults and juveniles in these natural forests is 1:1.

### 2.2. Genetic Analysis

We used the CTAB protocol to extract DNA from leaf tissues [45], and genotyped it using eight specified microsatellite loci [46]. We performed PCR in a LifePro thermal cycler from Bioer (Hangzhou, China) with a 13 mL mix containing 7.5 ng of genomic DNA, ultrapure water, buffer 1× (Fermentas Life Sciences, Burlington, ON, Canada) (3.25 ng of BSA, 3.25 mM of dNTPs, and 20 mM of MgCl2), forward and reverse primers with M13 tail (3.9 µM) marked with NED TM, 6-FAM TM, PET^®^ or VIC^®^, and 1U of Taq polymerase. The PCR followed the conditions: 94 °C for 3:30 min; 30 cycles of 94 °C for 1 min; specific annealing temperature of each primer for 45 s [46]; 72 °C for 1 min; a final extension of 72 °C for 7 min. We then verified the PCR product with an ABI 3130XL genetic analyzer (Applied Biosystems, Foster City, CA, USA) with GS500LIZ marker. We defined the size of the alleles by GeneMarker^®^ (Applied Biosystems, Foster City, CA, USA).

For each generation from each protected area we first estimated the allelic richness, observed heterozygosity (H_O_), and genetic diversity (H_E_) under the Hardy–Weinberg equilibrium. The level of inbreeding within a population was assessed by the inbreeding coefficient (f). The mean values of the allelic richness, H_E_, H_O_, and f were estimated considering a 95% confidence interval (95% CI) for all loci. Additionally, we calculated the genetic structure for adults and juveniles in both conservation units considering the F-statistics. The fixation index f and differentiation index θ were determined by performing 1000 permutations for all loci with Bonferroni sequential correction for multiple comparisons (α = 0.05). The results of all parameters were estimated by the FSTAT v.2.9.3.2 software [47].

We performed paternity analysis using the CERVUS 3.0.7 software [48] by combining genotypes from both protected areas, i.e., 95 adults and 83 juveniles, and all genotypes were georeferenced. We also calculated the distance between parents and their alleged progeny. For this, we considered that all sampled adults would be potential parents. For each juvenile, we used the likelihood method to account for the possible parents with 95% confidence. The likelihood ratio was expressed by the LOD score, where a positive score indicates that the candidate tree is likely to be a true parent. Only those adults and juveniles were considered with at least 6 out of 8 loci genotyped in the analysis. Paternity test results were estimated according to 95% strict and 80% relaxed confidence levels. We also performed analysis of spatial genetic structure for each protected area, for both juvenile and adult populations, using the SPAGeDi 1.3 software [49], to estimate the coefficient of relatedness or genetic distance between populations using genotype data from both protected areas, calculated according to Loiselle et al. [50].

## 3. Results

We observed that when comparing the number of alleles (Na) between generations, adults in both protected areas showed higher values (EVC = 12.9 and UBR = 10.9) than juveniles (EVC = 9.9 and UBR = 9.9). We also found that adults from EVC and UBR had similar values for allelic richness (9.6 and 8.8, respectively), H_E_ (0.815 and 0.818, respectively), and H_O_ (0.720 and 0.736, respectively). Although the inbreeding coefficients (f) were also similar (EVC = 0.103 and UBR = 0.097), they were positive and significant for both populations (*p*-value ≤ 0.01). In general, our genetic diversity estimations showed no statistical significance, considering only adults from both protected areas. 

We also observed that juveniles of both protected areas presented similar values for allelic richness (EVC = 8.1 and UBR = 7.1), H_E_ (EVC = 0.731 and UBR = 0.729), and H_O_ (EVC = 0.463 and UBR = 0.560). However, the inbreeding coefficient was higher for juveniles (f = EVC = 0.366 and UBR = 0.231) than adults (EVC = 0.103 and UBR = 0.097). Moreover, we detected a significant difference (*p*-value ≤ 0.01) of the f-value for juveniles between EVC = 0.366 and UBR = 0.231 (Table 1). When comparing the genetic structure between generations, we found pronounced differentiation between the protected areas. Considering adults, we detected a lesser genetic structure between EVC and UBR (θ = 0.017; *p*-value ≤ 0.05), while juveniles showed accentuated differentiation between both areas (θ = 0.109; *p*-value ≤ 0.001).

Paternity analysis revealed that 21 (49%) juveniles whose parents were detected showed positive LOD scores. We identified seven juveniles (16%) whose parents were detected with 95% confidence level. The remaining trees (84%) had low probability of paternity. The juveniles with the same parent label indicated that selfing has occurred in the sampled areas. We also observed that the maximum distance between individuals was 13.0 and 5.28 km in UBR and EVC, respectively. Additionally, the maximum distance between juveniles and adults of different populations was 127.64 km.

We did not find any spatial genetic structure in any of the trees from both protected areas (see Figure S1 in Supplementary Material).

## 4. Discussion

We detected novel results on the patterns of genetic diversity of an endemic and threatened tropical tree species, *M. multifida*, in two protected areas of the Brazilian Atlantic Forest hotspot. Additionally, we contrasted the genetic diversity among adults and juveniles, revealing a strong decay in genetic variability between generations in both protected areas. This has occurred due to habitat loss and fragmentation of the forest remnants in the region, which has led to reduction in their populations, with negative effects on heterozygosity. We also found that in studied fragments of Atlantic Forest, juveniles showed greater genetic structure than adults, likely reflecting the responses of young life stages to forest fragmentation and isolation of the populations. Finally, paternity analysis revealed that most identified parents of juveniles are in the same protected area, indicating that gene flow occurs mainly over a small distance. Our findings, therefore, highlight the pervasive effects of historical deforestation and fragmentation on the genetic diversity of *M. multifida* populations, a native Atlantic Forest species present in the areas for a long period and hypothetically contemplating two periods (before and after fragmentation). 

Analyses of genetic diversity revealed low observed heterozygosity among juveniles of *M. multifida* inhabiting each surveyed area (Table 1). As for allelic richness, only small differences between generations were observed, which indicates the adaptative potential of the species [51]. Indeed, reduction of genetic parameters evaluated in juvenile in relation to adult trees within each protected area demonstrated that the progenies have inherited genes from a reduced set of remaining trees. In particular, low genetic diversity has also been observed in another Neotropical tree, *Podocarpus sellowii* (Klotz.), due to the small size of the remnant population [52]. Before the intense deforestation and fragmentation, which mainly occurred in the last century in Bahia [53], it is likely that alleles were frequently exchanged across the state’s southern region, because the Atlantic Forest was originally continuous. This potentially explains high genetic diversity among the adult population of *M. multifida.* Indeed, adult trees often show responses of past landscape conditions on their genetic diversity [54,55,56].

A myriad of anthropogenic disturbances including deforestation, fragmentation, and overexploitation have likely affected the remaining populations of *M. multifida* in the studied region, since this species has been massively logged due to its high timber value [46]. The synergistic effect of these anthropogenic activities potentially led to reduction of the abundance of adult trees in forest fragments of southern Bahia, ultimately affecting genetic diversity in recent populations (i.e., juveniles). According to a recent study [57], the negative impacts of habitat loss on genetic diversity are observed in the various taxonomic group. In particular, low genetic diversity in juveniles observed in our study cannot be attributed to allelic richness, which is good news for the conservation of *M. multifida* future generations [58]. Apart from habitat loss and fragmentation, the reduction of genetic diversity depends on factors including the absence or inefficacy of dispersers or pollinators and the reduced effective population size [30,59].

We found inbreeding of both adults and juveniles inhabiting both protected areas. However, juveniles belonged to the smallest gene pool that remained in the populations after human disturbances. Additionally, despite the fact that no spatial genetic structure was found in the sampled populations, high inbreeding values might be related to historical aggregated crossing between individuals due to small-scale dispersion, a phenomenon also observed in the Amazon Forest for *Manilkara huberi* (Ducke) [44]. We observed inbreeding even in adults, demonstrating a process of natural inbreeding in the past, perhaps due to selective logging [44,60]. Other studies have confirmed similar processes, such as in juveniles of *Prunus africana* (Hook.f.) Kalkman in Kenya’s Kakamega Forest [60], and in adults of *Swietenia macrophylla* King in the Brazilian Amazon [61]. Inbreeding, also, can occur due to natural and biological factors related to pollination. However, there are still no reports on the pollen or seed dispersal syndrome of *M. multifida*. Studies in this respect exist in the Amazon Forest, revealing the interaction of *M. huberi* with bees of the genera *Melipona*, *Trigona*, *Plebeia*, *Tetrapedia*, and *Augochloropsis* [62]. Thus, we believe that the limitation of dispersal and/or pollination is a key factor related to our results. These bee genera are also found in the Atlantic Forest in southern Bahia [63], suggesting the possible interaction of *M. multifida* with these pollinators. As for the potential dispersers [38], reported that other species of the genus *Manilkara* interact with the golden-headed lion tamarin (*Leontopithecus chrysomelas*) in our region, given that species from this genus provide resources such as fruit and nectar [38].

Regarding population genetic structure, our results revealed that the differentiation of adult populations between the two protected areas is lower than that of juveniles. Despite the possible existence or homoplasy reported by Angers et al. (2000) [64], our results can be interpreted as indicating a difference in genetic structure between adults and juveniles, since it is hard to find the same spurious result in two generations, even when genotyping eight microsatellite loci [63]. This result can be associated with the possible migration events that occurred in the Pleistocene within the Bahia refuge [65]. These events led to the expansion of an ancestral gene pool along with the dense ombrophilousforest. However, forest fragmentation has affected *M. multifida* populations, isolating their individuals inside Atlantic Forest remnants. Other tropical trees have been found to have the same increase in differentiation index over a generation, such as the palm *Euterpe edulis* [66]. The decrease of genetic structure over generations is due to the adaptive potential of juveniles against the impact of habitat loss or population management in protected areas. Our results of paternity tests also corroborate that potential pollinators and seed dispersers are not effective in promoting long term gene flow, and thus make a small contribution to reduction of differentiation among the studied populations of *M. multifida* [30].

Our paternity analyses corroborated the inbreeding results, since most juveniles descended from adult trees inhabiting the same protected area (Table 2). Furthermore, these results confirm our initial hypothesis that gene flow occurs only over short distances, and indicate the pervasive effects of human disturbances on biodiversity [67,68]. In addition, other factors can decrease the gene flow, such as the propagule dispersal ability of species [68]. Short-distance gene flow has been observed in many trees based on their paternity analysis [69], such as *Prunus mahaleb* [70] and *Araucaria angustifolia* [71]. Moreover, the decline in gene flow distance of *E. edulis* was due to habitat loss [72]. The possible explanation for short-distance flow is the action of pollinators or dispersers. However, some of the juveniles established flow over the 127.64 km that separates the two fragments. However, since the Atlantic Forest was continuous in the past, the gene flow likely occurred between the two fragments studied.

## 5. Conclusions and Conservation Implications

Our results show that the *M. multifida* populations in the two protected areas still contain high levels of allelic richness. Nevertheless, anthropogenic disturbances have led to a reduction of heterozygosity in juveniles. This situation requires attention, since the diversity of the population can decline further over time even in the presence of adaptive potential to survive. Our results call attention to the importance of mitigation actions to contain deforestation in this region, since it is a key conservation area within the Atlantic Forest biodiversity hotspot. The low genetic diversity can be caused by the reduced effective population size of juveniles, which indicates the need to implement measures to improve habitat protection and expand forest cover via restoration projects in severely deforested landscapes.

The inbreeding and high genetic structure caused by isolation can generate significant constraints on population sizes. The two protected areas should be appropriately managed to conserve high genetic diversity, which can help maximize conservation of the regional diversity of *M. multifida*. The choice native species that are ecologically appropriate and genetically diverse is an important part of rehabilitating degraded habitats. We evaluated whether populations of *M. multifida* growing in two protected areas had the desired genetic diversity, and thus can form a viable population likely to persist for a long time in a degraded landscape. Further studies are needed to understand the mating pattern of *M. multifida* to confirm the exact reasons for the restricted gene flow. However, we suggest that this species can be further used for restoration programs in threatened Atlantic Forest fragments.

## Figures and Tables

**Table 1:** Genetic indices estimated for adult and juveniles of *M. multifida* trees recorded in the Una Biological Reserve (UBR) and Veracel Station (EVC). Allele number (Na), allelic richness (Ar), genetic diversity (H_E_), observed heterozygosity (H_O_), inbreeding coefficient (f), and differentiation index (θ).

	Site	Samples	Na	Ar	H_E_	H_O_	f	Θ
Adults	EVC	50	12.9	9.6	0.815	0.720	0.103 **	0.017 *
	UBR	45	10.9	8.8	0.818	0.736	0.097 **	
Juveniles	EVC	50	9.9	8.1	0.731	0.463	0.366 **	0.109 ***
	UBR	33	8.1	7.1	0.729	0.560	0.231 **	

* *p*-value ≤ 0.05, ** *p*-value ≤ 0.01, and *** *p*-value ≤ 0.001.

**Table 2:** Paternity analysis of *M. multifida* considering all sampled individuals in two protected areas in southern Bahia, Brazil.

Juvenile ID	Parent 1 ID	Parent 2 ID	LOD Score	TRIO Confidence	Distance Juveniles-P1 (km)	Distance Juveniles-P2 (km)	Distance P1-P2 (km)
EVC-J08	EVC-A43	EVC-A43	3.96	*	4.81	-	-
UBR-J11	UBR-A35	UBR-A35	7.54	*	13.0	-	-
EVC-J15	UBR-A32	UBR-A32	4.50	*	127.64	-	-
UBR-J30	UBR-A43	UBR-A43	2.90	*	-	-	-
UBR-J34	EVC-A113	UBR-A31	3.81	*	-	-	-
EVC-J35	EVC-A21	EVC-A21	4.51	*	5.28	-	-
EVC-J45	EVC-A39	EVC-A21	2.99	*	0.21	5.24	5.03
EVC-J02	EVC-A122	EVC-A30	1.43	+	-	5.19	-
EVC-J04	EVC-A43	EVC-A43	2.15	+	4.92	-	-
EVC-J13	EVC-A118	UBR-A40	2.35 × 10^−1^	+	-	-	-
UBR-J15	UBR-A45	UBR-A46	2.26	+	-	-	-
EVC-J16	EVC-A118	UBR-A23	8.33 × 10^−1^	+	-	127.58	-
UBR-J16	EVC-A28	EVC-A28	5.98 × 10^−1^	+	123.48	-	-
EVC-J22	UBR-A33	EVC-A118	2.19	+	127.46	-	-
UBR-J22	UBR-A46	EVC-A122	8.39 × 10^−2^	+	-	-	-
EVC-J29	UBR-A37	EVC-A22	2.31 × 10^−1^	+	-	5.28	-
EVC-J30	EVC-A59	UBR-A43	3.82 × 10^−1^	+	-	-	-
UBR-J31	EVC-A122	EVC-A113	7.59 × 10^−1^	+	-	-	-
EVC-J32	UBR-A28	UBR-A34	1.76	+	124	-	-
EVC-J43	EVC-A21	EVC-A21	1.78	+	5.24	-	-
EVC-J49	EVC-A122	UBR-A26	1.70	+	-	-	-

An asterisk (*) indicates that the confidence level is 95% using the “strict” criterion implemented in CERVUS, (+) Indicates that the confidence level is 80% using the “relaxed” criterion implemented in CERVUS, LOD = logarithm of odds. LOD scores at the strict confidence level of 99%. A = Adults and J = Juveniles.

## Data Availability

Data are available on request to the corresponding author.

## Supplementary Materials

The following are available online at https://www.mdpi.com/article/10.3390/genes12122025/s1, Figure S1: Fine-scale spatial genetic structure of *M. multifida* adult and juvenile populations in two protected forests in Brazil.
