# Supplementary material for: Gene Flow and Genetic Structure Reveal Reduced Diversity between Generations of a Tropical Tree, Manilkara multifida Penn., in Atlantic Forest Fragments
Source: Genes (Basel). 2021 Dec 20;12(12):2025. doi: 10.3390/genes12122025 (PMC8701937; doi:10.3390/genes12122025)
Supplement: Supplementary file 1 [file genes-12-02025-s001.zip › genes-1447544-supplementary-Figure S1.pdf]

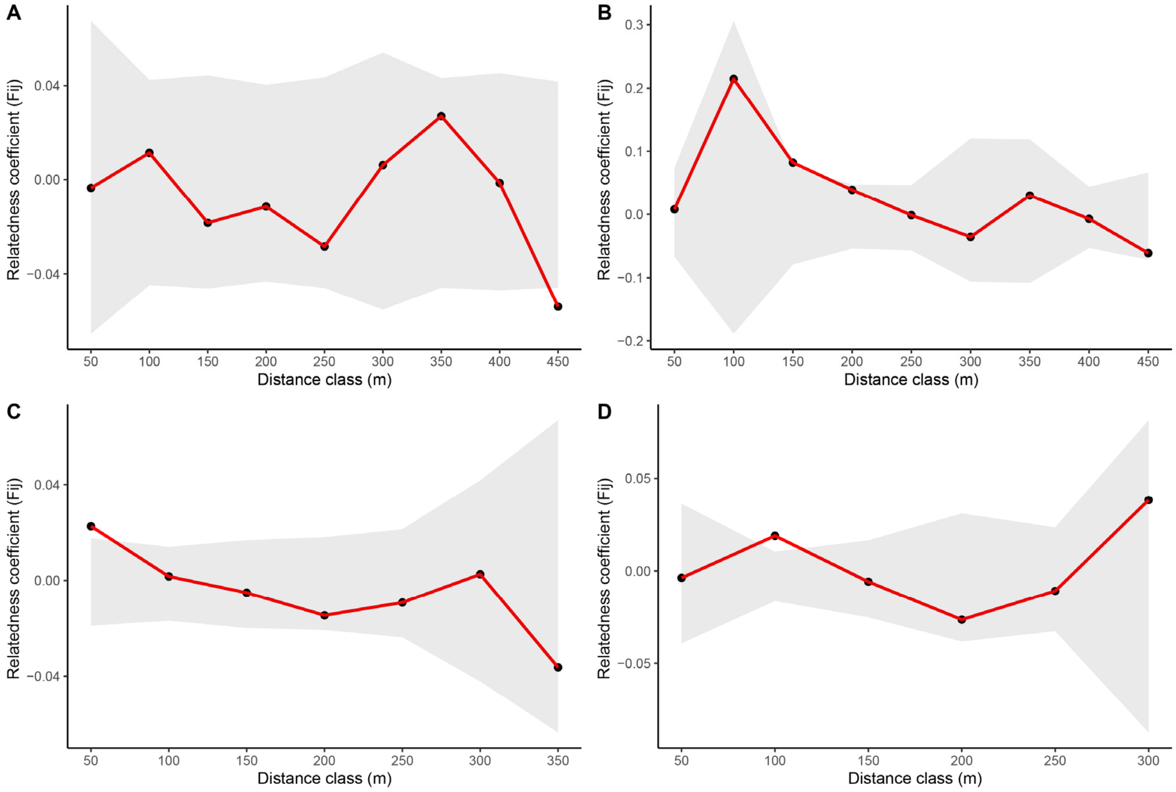

Figure S1. Fine-scale spatial genetic structure of *M. multifida* adult and juvenile populations in two protected forests in Brazil.
